# Pharmacolgy teaching: Need for a sea change

**DOI:** 10.4103/0253-7613.59930

**Published:** 2009-12

**Authors:** Vishal Sharma, Rashmi Sharma

**Affiliations:** Department of Medicine, University College of Medical Sciences, Delhi, India. E-mail: docvishalsharma@gmail.com; 1 Intern, BJS Dental College, Ludhiana, India

Sir,

One cannot but agree with the pitch for substantial changes in the current pharmacology curriculum.[1] Traditional teaching of pharmacology has encouraged rote learning and has taken away the interest of students from what can be a very interesting subject. The practical teaching has been even worse, with emphasis being on ancient techniques. As a student, I never understood the need to perform those experiments on rabbit intestines and the need to prepare those tinctures and lotions that I no longer remember. Training in practical clinical pharmacology was extremely limited. Any change in this archaic curriculum needs all the support from teachers, policy makers and administrators.

Current medical graduates learn their first lessons in rational and appropriate prescribing in busy out patient departments during their internship. Their knowledge about practical drug dosing and modifications to be made in various clinical scenarios leaves much more to be desired. The new guidelines for teaching pharmacology must be radical in scraping what is useless and in recommending a practical patient-based learning. This will bring the interest of medical students back into pharmacology classes.
